# Clot Retention and Advanced Age as Predictors of Intervention in Patients Presenting With Gross Hematuria: A Retrospective Cohort Study

**DOI:** 10.7759/cureus.114056

**Published:** 2026-08-06

**Authors:** Abdalhakim S Nimate, Sofian F AlBdour, Belal M Abunaja, Zaid M Alqudah, Hala A AbuQamer

**Affiliations:** 1 Urology, King Hussein Medical City, Amman, JOR; 2 Radiology, King Hussein Medical City, Amman, JOR

**Keywords:** bladder tumor, gross hematuria, invasive intervention, predictors of intervention, urinary tract infection, urologic emergency

## Abstract

Background and objective

Gross hematuria is a common urologic manifestation that often requires hospitalization and immediate evaluation. Early identification of factors associated with the need for intervention can aid in risk stratification and guide timely clinical management. The objective of this study was to evaluate hospitalized patients with gross hematuria and identify predictors associated with the need for intervention.

Methods

This retrospective cohort study included adult patients admitted with gross hematuria between January 2023 and December 2025 at Prince Hussein Urology Center, Amman, Jordan, affiliated with the Royal Medical Services. Data were obtained from the Hakeem electronic medical record system. A total of 330 patients were included and categorized into an intervention group (n = 99; 30%) and a nonintervention group (n = 231; 70%). Univariable analysis followed by multivariable logistic regression was performed to identify predictors of intervention. A p-value < 0.05 was considered statistically significant.

Results

Among 330 patients admitted with gross hematuria, 99 (30.0%) required urologic intervention. Patients requiring intervention were older (mean age, 66.8 ± 14.9 years) and more frequently had clot retention, anemia, and elevated serum creatinine levels. Bladder tumor was the most common etiology, accounting for 39.4% of cases in the intervention group. On multivariable analysis, clot retention (OR, 4.11; 95% CI, 2.23-7.59; p < 0.001) and increasing age (OR, 1.02; 95% CI, 1.00-1.04; p = 0.028) were independently associated with the need for urologic intervention after multivariable adjustment.

Conclusions

Clot retention and advancing age are independent predictors of urologic intervention in patients admitted with gross hematuria. Early recognition of these factors may help guide risk stratification and facilitate timely management.

## Introduction

Hematuria, defined as the presence of blood in the urine, is a common clinical finding in urological practice and accounts for more than 20% of all urological assessments. It may be classified in various ways, including intermittent or persistent, glomerular or non-glomerular, and symptomatic or asymptomatic; however, in everyday clinical practice, the most useful distinction is between gross (visible) and microscopic (non-visible) hematuria [1].

The etiology of gross hematuria is diverse and closely related to patient age. In younger individuals, it is more commonly associated with benign conditions such as urolithiasis, UTIs, and trauma. In contrast, in older patients, particularly those over 50 years of age, malignancy becomes a major concern, with bladder cancer being the most frequent cause, followed by renal and prostate malignancies. Benign prostatic hyperplasia also contributes significantly to this group [2].

Clinically, gross hematuria has been reported to occur in approximately 2.5% of the population. In a review of observational studies, the estimated annual rates of fatal bleeding, major bleeding, and overall bleeding were 0.8%, 4.9%, and 15%, respectively. Gross hematuria may result in clot formation and urinary retention, requiring prompt management. Initial treatment typically involves bladder decompression, continuous bladder irrigation, and clot evacuation. However, a subset of patients fails conservative management and requires invasive intervention, including endoscopic, surgical, or radiological procedures [3].

Despite its clinical importance, evidence guiding the early identification of patients who require invasive intervention remains limited. In particular, predictors of intervention among hospitalized patients with gross hematuria remain poorly defined [4]. Therefore, this study aimed to identify clinical and laboratory predictors associated with the need for specialized urologic intervention among patients admitted with gross hematuria.

## Materials and methods

This retrospective cohort study was conducted at the Prince Hussein Urology Center, Amman, Jordan, affiliated with the Royal Medical Services. This single-center study was performed at a tertiary referral urology center. Adult patients admitted with gross hematuria between January 2023 and December 2025 were included. The index time point was defined as the time of hospital admission. The primary outcome of the study was the requirement for advanced urologic intervention during the same hospital admission.

Ethical approval for this study was obtained from the Royal Medical Services Ethical Committee prior to data collection. The study was conducted in accordance with the principles of the Declaration of Helsinki. The requirement for informed consent was waived because of the retrospective nature of the study. The Hakeem electronic medical record system, the national electronic medical record platform in Jordan, was used to extract data retrospectively. Patient identifiers were removed during data retrieval to maintain confidentiality. Data extraction was performed by trained investigators using a standardized data collection form. The study was reported in accordance with the Strengthening the Reporting of Observational Studies in Epidemiology (STROBE) guidelines.

A total of 330 patients met the inclusion criteria and were included in the final analysis. Patients were categorized according to the primary outcome into two groups: Group I, patients who required urologic intervention during the same hospital admission (n = 99, 30%); and Group II, patients managed conservatively without intervention (n = 231, 70%).

Sample size estimation was performed using a single-proportion formula, assuming an anticipated intervention rate of 30% based on previous studies. With a 95% confidence level and a 5% margin of error, the minimum required sample size was calculated to be approximately 323 patients. The final cohort of 330 patients exceeded this requirement and was therefore sufficient for multivariable logistic regression analysis, consistent with the recommended minimum of 10 outcome events per predictor variable.

Patients were identified through hospital admission records. All consecutive eligible patients admitted during the study period were included to minimize selection bias. Eligible patients were adults aged ≥18 years admitted for evaluation or management of gross hematuria through the emergency department, referral from peripheral hospitals, or direct admission from the urology outpatient clinic. Indications for hospital admission included persistent gross hematuria, clot retention, symptomatic anemia, hemodynamic instability, urinary retention, or the need for inpatient diagnostic or therapeutic management.

Exclusion criteria included microscopic hematuria, patients managed entirely in the outpatient setting, hematuria occurring within 30 days following urologic surgery, and incomplete medical records.

Demographic and clinical variables collected included age, sex, anticoagulant use, hypertension, diabetes mellitus, coronary artery disease, chronic kidney disease, hemoglobin level, and serum creatinine level on admission. Clinical features, including clot retention (defined as urinary obstruction due to blood clots in the urinary bladder requiring catheterization, bladder irrigation, or endoscopic evacuation [5]) and UTI (defined based on clinical symptoms supported by positive urinalysis and/or urine culture [6]), were also recorded. Laboratory values obtained at admission were used for analysis. Continuous variables were additionally analyzed as categorical variables using clinically relevant cutoff values to enhance clinical interpretability and facilitate risk stratification. Low hemoglobin was defined as hemoglobin <10 g/dL [7], and elevated serum creatinine was defined as serum creatinine >1.5 mg/dL [8].

The underlying etiology of hematuria was determined according to the final documented diagnosis and categorized as bladder tumor, benign prostatic hyperplasia-related bleeding, UTI, urolithiasis, upper tract urothelial carcinoma, anticoagulant-related bleeding, prostate cancer, trauma or instrumentation, or idiopathic hematuria when no specific cause was identified.

All patients underwent an initial clinical evaluation, including a detailed medical history, physical examination, laboratory investigations, and imaging studies when indicated. Initial management typically included placement of a large-bore 24-Fr three-way Foley catheter with continuous bladder irrigation using normal saline in patients with active bleeding or clot retention. Manual irrigation and clot evacuation were performed when necessary to relieve urinary retention caused by intravesical clots. Additional diagnostic evaluation included ultrasonography or computed tomography of the urinary tract, followed by cystoscopic evaluation when indicated. Management decisions followed standard urological practice.

Urologic intervention was defined as any endoscopic or radiologic procedure performed during the same hospital admission to control bleeding or treat the underlying cause of hematuria [9]. These procedures included cystoscopic clot evacuation, transurethral resection of bladder tumor (TURBT), transurethral resection of the prostate (TURP), endoscopic fulguration of bleeding lesions, ureteroscopy for stone management, percutaneous nephrostomy or ureteral stenting, and angioembolization. The decision to perform urologic intervention was made by the treating urologist based on clinical judgment and predefined clinical indications, including failure of conservative management, persistent or recurrent clot retention, hemodynamic instability, a significant hemoglobin decrease requiring transfusion, or identification of an underlying pathology requiring procedural management.

Blood transfusion was administered to patients with symptomatic anemia, ongoing bleeding with hemodynamic compromise, or hemoglobin levels below 7-8 g/dL according to accepted transfusion thresholds [10]. Additional in-hospital outcomes recorded included blood transfusion, ICU admission, in-hospital mortality, and length of hospital stay.

Several methodological steps were undertaken to minimize potential sources of bias. Selection bias was minimized by including all consecutive eligible patients during the study period. Information bias was minimized through standardized data extraction using predefined variable definitions. Missing data were minimal and were handled using complete-case analysis; therefore, no imputation was performed.

Statistical analyses were performed using IBM SPSS Statistics for Windows, version 26.0 (released 2018; IBM Corp., Armonk, NY, USA). Continuous variables were expressed as mean ± SD, while categorical variables were expressed as frequencies and percentages. The distribution of continuous variables was assessed using the Shapiro-Wilk test to evaluate normality. Because the data were approximately normally distributed, comparisons between groups were performed using the independent Student’s t-test for continuous variables and the chi-square test for categorical variables. Continuous variables were also analyzed as categorical variables using clinically relevant cutoff values to enhance clinical interpretability and facilitate risk stratification. Univariable logistic regression analysis was performed to identify variables associated with the need for urologic intervention. Variables with p < 0.20 in the univariable analysis, along with clinically relevant variables, were included in the multivariable logistic regression model to reduce confounding, based on clinical relevance and prior literature. Results are reported as ORs with 95% CIs. Multicollinearity among predictor variables was assessed using the variance inflation factor, and no significant collinearity was detected. Model goodness-of-fit was assessed using the Hosmer-Lemeshow test. Potential interactions between key variables were explored and were not statistically significant. A two-tailed p-value < 0.05 was considered statistically significant.

## Results

The analysis included a total of 330 patients admitted with gross hematuria. Of these, 99 patients (30.0%) required urologic intervention, whereas 231 patients (70.0%) were managed conservatively.

As shown in Table 1, the baseline demographic and clinical characteristics are summarized. Unsurprisingly, patients who required intervention were older than those managed conservatively. The intervention group also demonstrated higher rates of anticoagulant use, low hemoglobin levels (<10 g/dL), elevated serum creatinine (>1.5 mg/dL), clot retention, and UTI. No statistically significant differences were observed between the two groups in terms of sex distribution or comorbidities, including hypertension, diabetes mellitus, coronary artery disease, and chronic kidney disease.

**Table 1:** Baseline demographic and clinical characteristics.

Variable	Intervention (n = 99)	No intervention (n = 231)	p-value
Age, years (mean ± SD)	66.8 ± 14.9	59.2 ± 15.6	<0.001
Male sex, n (%)	70 (70.7)	150 (64.9)	0.372
Chronic kidney disease	12 (12.1)	21 (9.1)	0.522
Hypertension	48 (48.5)	103 (44.6)	0.596
Coronary artery disease	16 (16.2)	38 (16.5)	1.000
Diabetes mellitus	30 (30.3)	64 (27.7)	0.729
Anticoagulant use	34 (34.3)	52 (22.5)	0.035
Low hemoglobin (<10 g/dL)	38 (38.4)	42 (18.2)	<0.001
Serum creatinine >1.5 mg/dL	22 (22.2)	28 (12.1)	0.029
Clot retention	35 (35.4)	25 (10.8)	<0.001
UTI	18 (18.2)	22 (9.5)	0.043

The distribution of underlying etiologies is presented in Table 2. Bladder tumor was the most common cause of hematuria, particularly among patients requiring intervention. Other causes included UTI, urolithiasis, benign prostatic hyperplasia-related bleeding, and idiopathic hematuria.

**Table 2:** Etiology of gross hematuria.

Etiology	Intervention (n = 99)	No intervention (n = 231)	p-value
Bladder tumor	39 (39.4)	46 (19.9)	<0.001
Benign prostatic hyperplasia	9 (9.1)	34 (14.7)	0.225
UTI	18 (18.2)	22 (9.5)	0.043
Urolithiasis	13 (13.1)	32 (13.9)	1.000
Upper tract urothelial carcinoma	4 (4.0)	7 (3.0)	0.894
Anticoagulation-related bleeding	8 (8.1)	28 (12.1)	0.375
Prostate cancer	3 (3.0)	6 (2.6)	1.000
Trauma/instrumentation	2 (2.0)	9 (3.9)	0.592
Idiopathic	3 (3.0)	47 (20.3)	<0.001

In-hospital outcomes are shown in Table 3. Patients undergoing intervention had significantly higher rates of blood transfusion. However, there were no statistically significant differences between the groups with respect to ICU admission, in-hospital mortality, or length of hospital stay.

**Table 3:** In-hospital outcomes.

Outcome	Intervention (n = 99)	No intervention (n = 231)	p-value
Blood transfusion, n (%)	30 (30.3)	18 (7.8)	<0.001
ICU admission, n (%)	4 (4.0)	16 (6.9)	0.450
In-hospital mortality, n (%)	1 (1.0)	5 (2.2)	0.787
Length of stay, days (mean ± SD)	4.7 ± 2.0	5.0 ± 2.0	0.312

Among the 99 patients who underwent advanced intervention, the types of procedures performed are detailed in Table 4. Cystoscopic clot evacuation was the most frequently performed procedure, followed by TURBT. Other interventions included ureteroscopy for stone management, TURP, endoscopic fulguration, percutaneous nephrostomy or ureteral stenting, and angioembolization.

**Table 4:** Types of urologic interventions (n = 99). TURBT, transurethral resection of bladder tumor; TURP, transurethral resection of the prostate

Procedure	Number (%)
Cystoscopic clot evacuation	35 (35.4)
TURBT	25 (25.3)
TURP	3 (3.0)
Endoscopic fulguration	6 (6.1)
Ureteroscopy (stone treatment)	15 (15.2)
Percutaneous nephrostomy/ureteral stenting	10 (10.1)
Angioembolization	3 (3.0)
Other	2 (2.0)

In the univariable logistic regression analysis (Table 5), increasing age, anticoagulant use, low hemoglobin level, elevated serum creatinine, clot retention, and UTI were significantly associated with the need for urologic intervention.

**Table 5:** Univariable logistic regression analysis.

Variable	OR	95% CI	p-value
Age	1.03	1.02-1.05	<0.001
Male sex	1.3	0.78-2.17	0.309
Chronic kidney disease	1.38	0.65-2.93	0.402
Hypertension	1.17	0.73-1.87	0.515
Coronary artery disease	0.99	0.52-1.88	0.978
Diabetes mellitus	1.13	0.68-1.90	0.632
Anticoagulant use	1.8	1.07-3.02	0.026
Low hemoglobin (<10 g/dL)	2.8	1.66-4.74	<0.001
Serum creatinine (>1.5 mg/dL)	2.07	1.12-3.84	0.021
Clot retention	4.51	2.51-8.09	<0.001
UTI	2.11	1.08-4.14	0.030

Variables meeting the predefined inclusion criteria were entered into the multivariable logistic regression model (Table 6). After adjustment, clot retention remained the strongest independent predictor of intervention, demonstrating the highest magnitude of association among all evaluated variables (OR 4.11, 95% CI 2.23-7.59, p < 0.001). Increasing age was also independently associated with intervention (OR 1.02, 95% CI 1.00-1.04, p = 0.028). Other variables, including anticoagulant use, low hemoglobin level, elevated serum creatinine, and UTI, were not independently associated with intervention after adjustment.

**Table 6:** Multivariable logistic regression analysis.

Variable	Adjusted OR	95% CI	p-value
Age	1.02	1.00-1.04	0.028
Anticoagulant use	1.26	0.69-2.30	0.461
Low hemoglobin (<10 g/dL)	1.65	0.91-2.99	0.102
Serum creatinine (>1.5 mg/dL)	1.9	0.96-3.76	0.064
Clot retention	4.11	2.23-7.59	<0.001
UTI	1.2	0.57-2.56	0.629

## Discussion

Nearly 30% of the patients in this retrospective cohort who were admitted with gross hematuria required urologic intervention, implying that a substantial proportion of hospitalized cases represent clinically significant conditions rather than self-limited occurrences. This finding is consistent with current evidence and guideline recommendations emphasizing the need for thorough evaluation of gross hematuria because of its strong association with underlying pathology [11].

Regarding etiology, bladder tumors were the most frequent cause among patients requiring intervention in our cohort. This finding is consistent with previous literature, in which visible hematuria is the most common presenting symptom of bladder cancer. As emphasized by Babjuk et al., early identification of malignant causes remains a cornerstone of the diagnostic evaluation of hematuria [12], highlighting the importance of maintaining a high index of suspicion, particularly in older patients.

The proportion of patients requiring intervention in our study is consistent with prospective observational data. Tan et al. demonstrated that patients presenting with visible hematuria often harbor significant urological disease requiring further evaluation, particularly in the presence of additional clinical risk factors [4].

A thorough diagnostic workup is of paramount importance, as highlighted by Bolenz et al., who emphasized that following standardized diagnostic protocols facilitates the identification of clinically important pathologies in hospitalized patients presenting with hematuria [13]. Our findings support a structured, guideline-based approach to evaluation.

Older age was independently associated with the need for intervention in our analysis. This finding is consistent with previous studies showing that advancing age is strongly associated with a higher likelihood of significant urological pathology, particularly malignancy [14]. In contrast, common comorbidities, including hypertension, diabetes mellitus, and coronary artery disease, were not associated with intervention, suggesting that the need for procedural management is more likely to be driven by disease-specific factors than by baseline patient comorbidities [15].

Similarly, serum creatinine was not an independent predictor of intervention in our study. Although it showed an association in the univariable analysis, this relationship was not sustained after multivariable adjustment. Comparable findings have been reported by Ragonese et al., who demonstrated that clinical and hemodynamic factors carry greater predictive value than baseline renal function alone [16]. This suggests that the decision to intervene is more closely related to the severity and source of bleeding than to renal impairment.

Clot retention emerged as the strongest independent predictor of intervention in our cohort. This finding is clinically intuitive, as clot retention reflects active bleeding with urinary obstruction and failure of conservative management. Hicks and Li similarly reported that macroscopic hematuria associated with clot formation frequently necessitates urgent intervention to relieve obstruction and stabilize the patient [17].

Although low hemoglobin levels were associated with intervention in the univariable analysis, this association did not persist after adjustment. This suggests that anemia may reflect the severity of bleeding but does not independently determine the need for procedural intervention. Similar observations have been reported in previous studies evaluating outcomes in patients with urological malignancies [18].

Although anticoagulant use was associated with hematuria in the univariable analysis, it did not independently predict intervention in our cohort. Previous studies have shown that anticoagulation increases the risk of hematuria-related complications and health care resource utilization [19]. Anticoagulant use therefore appears to be a contributing factor rather than a primary determinant of intervention. Additionally, hematuria in anticoagulated patients may reveal an underlying urological pathology [20].

UTI was associated with presentation but not with intervention in our analysis. This finding suggests that although infection may contribute to hematuria, it is less likely to necessitate invasive management. Moreover, the presence of infection does not exclude underlying malignancy, and patients should still undergo appropriate evaluation, as highlighted in previous reports [21].

In summary, our findings show that gross hematuria is a heterogeneous clinical condition in which the need for intervention appears to be primarily influenced by signs of active bleeding and underlying urological pathology rather than by comorbidities or secondary contributing factors. Recognizing this distinction is crucial for timely risk stratification and informed clinical decision-making.

Strengths of this study include the relatively large cohort of hospitalized patients and its reflection of real-world clinical practice at a tertiary referral center. The use of standardized data collection and multivariable analysis allowed adjustment for potential confounders. However, several limitations should be acknowledged. The retrospective design introduces the potential for selection and information bias. Additionally, the single-center setting may limit generalizability, and management decisions were based on clinical judgment, which may have introduced variability in intervention thresholds. Residual confounding cannot be completely excluded.

## Conclusions

One in three patients admitted with gross hematuria in this cohort required urologic intervention, highlighting the clinical significance of these presentations. Clot retention was the strongest predictor of intervention, while advancing age also independently increased the likelihood of requiring procedural management. These findings indicate that the need for intervention is primarily dictated by the severity of bleeding and underlying pathology rather than by comorbid conditions. Early recognition of these factors may facilitate risk stratification and inform clinical decision-making in hospitalized patients.
